# Evaluation of vitamin D status, vitamin D receptor expression, and innate immune mediators in COVID-19

**DOI:** 10.3389/fendo.2025.1600623

**Published:** 2025-08-19

**Authors:** Ferdos Missilmani, Dima Maarabouni, Elie Salem-Sokhn, Spyridon N. Karras, Hana M. A. Fakhoury, Said El Shamieh

**Affiliations:** ^1^ Molecular Testing Laboratory, Department of Medical Laboratory Sciences, Faculty of Health Sciences, Beirut Arab University, Beirut, Lebanon; ^2^ Laboratory of Biological Chemistry, Medical School, Aristotle University, Thessaloniki, Greece; ^3^ College of Medicine, Alfaisal University, Riyadh, Saudi Arabia

**Keywords:** COVID-19, vitamin D, *VDR*, innate immunity, inflammatory biomarkers

## Abstract

**Background and objectives:**

The Coronavirus disease 2019 (COVID-19) pandemic underscored the importance of identifying host factors that influence susceptibility to infection. Vitamin D signaling, mediated via its receptor (*VDR*), along with innate immune mediators such as antimicrobial peptides (e.g., *DEFA1-3*) and inflammatory chemokines (e.g., *CCL20*), plays a critical role in antiviral defense. This study aimed to determine how serum vitamin D status and gene expression of *VDR*, *DEFA1-3*, and *CCL20* associate with COVID-19 risk in a Lebanese cohort.

**Methods:**

This prospective observational study assessed serum vitamin D concentrations and nasopharyngeal gene expression in Lebanese participants tested for SARS-CoV-2 between January and March 2024. We enrolled 264 patients undergoing RT-qPCR (targeting *ORF1*, *N*, and *E* genes) and quantified serum 25-hydroxyvitamin D [25(OH)D]. In a subset of 70 individuals stratified by COVID-19 status, we measured *VDR*, *DEFA1-3*, *CCL20*, and *GAPDH* expression by RT-qPCR. Multiple logistic regression and Pearson correlation analyses were performed.

**Results:**

Serum vitamin D levels and *CCL20* expression were not significantly associated with COVID-19 status. Elevated *VDR* expression in nasopharyngeal tissue correlated with lower COVID-19 risk (OR = 0.40, *p* = 0.05) and inversely with 25(OH)D levels (r = –0.61, *p* = 0.04). Higher *DEFA1–3* expression reduced COVID-19 risk by 81.6% (OR = 0.184, *p* = 0.012). Among COVID-19 negatives, *VDR* correlated with *CCL20* (r = 0.59, *p* < 0.01); among positives, *VDR* correlated with *DEFA1-3* (r = 0.45, *p* < 0.05).

**Conclusion:**

Our findings reveal a complex interplay between systemic vitamin D status, local VDR expression, and innate inflammatory mediators in COVID-19. They support a model in which both micronutrient levels and tissue-specific vitamin D signaling modulate host susceptibility and disease severity.

## Introduction

The Coronavirus disease 2019 (COVID-19) pandemic has prompted extensive investigation into host factors that influence susceptibility and disease severity (1). Among these factors, vitamin D has garnered considerable attention due to its immunomodulatory properties (2–6). Early observational studies highlighted a potential protective role for vitamin D, as deficiency was frequently associated with increased disease severity, hospitalization rates, and mortality in COVID-19 patients (7, 8). Vitamin D can impact numerous pathways in the host immune response, promoting an appropriate inflammatory reaction while suppressing an excessive one (5). Vitamin D’s immunomodulatory role is significant in COVID-19, where severe cases involve excessive innate immune activation and lung immunothrombosis (9). Its effects are mediated through the vitamin D receptor (*VDR*), expressed on macrophages, dendritic cells, T-cells, and respiratory epithelial cells (4, 10, 11). Activation of *VDR* by active vitamin D modulates gene transcription, enhancing both innate and adaptive immune responses to strengthen antimicrobial defense (4, 9, 10).

While systemic vitamin D status is commonly assessed via circulating serum 25-hydroxyvitamin D [25(OH)D] concentrations, recent research has suggested that local tissue responsiveness—reflected by *VDR* expression—may be equally, if not more, relevant for immune protection (12). Indeed, local receptor expression levels potentially indicate the ability of tissues to mount effective vitamin D-dependent immune responses better than serum vitamin D concentrations alone. However, the relationship between local *VDR* expression and systemic vitamin D status remains inadequately explored in the context of viral infections such as COVID-19.

In Lebanon, vitamin D deficiency remains widespread despite plentiful sunshine (13, 14). This is exacerbated by cultural clothing that limits sun exposure and by low dietary intake of vitamin D–rich foods (13, 14). Lebanon’s COVID-19 clinical management protocol, aligned with the World Health Organization’s 2023 guidelines, did not recommend routine vitamin D supplementation as part of standard treatment during the study period.

Innate inflammatory mediators such as the alpha-defensins (*DEFA1-3*), produced by neutrophils and mucosal cells, exhibit antiviral activity by disrupting viral membranes and blocking entry (15, 16), and are linked to reduced respiratory infections (17, 18). They also maintain immune homeostasis and epithelial integrity during infections (16, 19). Chemokines like *CCL20* recruit immune cells to infected tissues (20, 21). Elevated *CCL20* levels are associated with severe COVID-19 outcomes, including acute respiratory distress syndrome (ARDS) and multisystem inflammatory syndrome in children (MIS-C), indicating a pathogenic role (22–24).

This prospective observational study aimed to assess how serum 25(OH)D concentrations and nasopharyngeal expression of *VDR*, *DEFA1-3*, and *CCL20* are associated with COVID-19 status in Lebanese participants tested between January and March 2024.

## Materials and methods

### Study design and participants

The study was a prospective observational analysis conducted between January and March 2024, involving 264 adult participants who presented for measurement of serum 25(OH)D levels and/or severe acute respiratory syndrome coronavirus 2 (SARS-CoV-2) testing. Eligible participants were consecutively enrolled after meeting the inclusion and exclusion criteria at Lebanese Hospital Geitaoui, a tertiary center in Lebanon, during the Omicron BA.5 wave. Serum 25(OH)D levels were measured for all participants during the winter season (year 2024) to minimize the effect of seasonal variation on vitamin D concentrations. Participants were enrolled using a consecutive sampling strategy during the study period. A history of vitamin D supplementation within the past three months was recorded. In a subset of 70 patients, nasopharyngeal tissue was collected for gene expression analysis. Exclusion criteria included chronic autoimmune diseases, active malignancy, uncontrolled diabetes mellitus, chronic renal disease, and acute infections other than COVID-19.

### Ethical considerations

This study was conducted in full accordance with ethical guidelines and was approved by the Institutional Review Board (IRB) of Lebanese Hospital Geitaoui-UMC under protocol code 2024-IRB-010. Written informed consent was obtained from the study participants. No personally identifiable information was included in the analyses or subsequent reporting, ensuring that all data were anonymized and handled with the utmost confidentiality.

### Data collection and laboratory analyses

#### Serum vitamin D measurement

Venous blood samples were collected, and total serum 25(OH)D concentrations were quantified using the Roche Elecsys™ Vitamin D Total Assay. Participants were subsequently classified as vitamin D deficient (<20 ng/mL), insufficient (20–30 ng/mL), or sufficient (≥30 ng/mL) based on the Endocrine Society Clinical Practice Guidelines (25). The coefficient of variation for 25(OH)D measurement assays was between 3% to 8%.

#### RNA extraction and cDNA synthesis

Nasopharyngeal swab samples from a subset of 70 patients were processed for RNA extraction using the ANDiS Viral RNA Auto Extraction & Purification Kit in conjunction with the ANDiS 350 Automated Nucleic Acids Extraction System. In this automated protocol, viral particles were first lysed to release RNA, which was then captured on magnetic beads. Following washes to remove impurities, the RNA was eluted into a clean solution for further analysis. RNA concentration and purity were assessed using a NanoDrop spectrophotometer (Thermo Fisher Scientific), ensuring A260/A280 ratios between 1.8 and 2.0. The extracted RNA was quantified, stored at –80°C, and subsequently reverse-transcribed into complementary DNA (cDNA) using a commercial reverse transcription kit, following the manufacturer’s instructions.

#### SARS−CoV−2 detection

SARS-CoV-2 detection was performed using the ANDiS FAST SARS-CoV-2 Detection Kit (3D Biomedicine Science & Technology Co., Limited), targeting the *ORF1*, *N*, and *E* genes. Samples were collected 1–3 days post-symptom onset, between January and March 2024, during which the Omicron BA.5 variant predominated in Lebanon. Although the detection assay targeted conserved SARS-CoV-2 genes (ORF1, N, E), variant-specific genotyping was not performed. Each reaction contained 15 ng of extracted RNA, Positive and negative controls were included to validate the assay. Amplification was conducted on the Bio-Rad CFX96 Real-Time PCR System using the following thermal cycling conditions: reverse transcription at 50°C for 10 minutes, initial denaturation at 95°C for 3 minutes, followed by 40 cycles of denaturation at 95°C for 15 seconds and annealing/extension at 60°C for 30 seconds. Cycle threshold (Ct) values were determined for each gene target, with a Ct ≤40 in at least two of the three targets (*ORF1*, *N*, or *E*) considered positive. All individuals with positive RT-PCR tests also presented with clinical symptoms (fever, cough, myalgia, anosmia), and were considered as having symptomatic COVID-19. As for the control group (non-COVID-19), they were also tested due to the presence of similar respiratory symptoms, but their test results were negative for SARS-CoV-2.

#### Quantitative real−time PCR for gene expression

RT−qPCR was performed to quantify the expression of *VDR* and the inflammatory genes *DEFA1−3* and *CCL20* in nasopharyngeal tissue samples. Gene−specific primers were designed and validated for efficiency and specificity. Each 20 µL reaction mixture contained SYBR Green Supermix, optimized concentrations of forward and reverse primers, and 70 ng of cDNA template, with *GAPDH* serving as the housekeeping gene for normalization. The thermal cycling protocol commenced with an initial enzyme activation and denaturation step at 95°C for 3 minutes, followed by 40 cycles of denaturation at 95°C for 15 seconds and a combined annealing/extension step at 60°C for 30 seconds. All reactions were executed in duplicate to ensure reproducibility and accuracy.

### Statistical analysis

Analyses were performed using IBM SPSS Statistics. Continuous variables are expressed as the mean ± standard deviation, and categorical variables are presented as frequencies and percentages. Normality of continuous variables was assessed using the Kolmogorov-Smirnov test prior to applying parametric tests. Independent samples t-tests and Chi-square tests were used to compare continuous and categorical variables, respectively, between patients with and without COVID-19. The Ct values were used solely to define SARS-CoV-2 positivity as a binary variable. Quantitative Ct data were not included in the downstream correlation or regression analyses. The sample size was calculated using G*Power software based on a moderate effect size (Cohen’s d = 0.6), with α = 0.05 and power = 80%, resulting in a minimum of 45 participants per group.

For the entire cohort, a multiple logistic regression analysis was conducted, adjusting for age, sex, and BMI, to identify independent predictors of COVID-19 disease. The results are reported as odds ratios and 95% confidence intervals.

For the subset of 70 patients with gene expression data, separate logistic regression models, adjusted for age, sex, and BMI, were used to evaluate the association between the normalized expression of *VDR*, *DEFA1-3*, and *CCL20* and COVID-19 status. For each gene, a median value was calculated and further used as a cut-off to classify the gene expression as high or low. Pearson correlation analysis was used to assess the relationship between serum 25(OH)D concentrations and *VDR* expression, as well as the association between inflammatory biomarker levels and SARS-CoV-2 viral gene expression in COVID-19-positive patients. A two-tailed *p*-value ≤ 0.05 was considered statistically significant.

## Results

The study cohort consisted of 264 patients, comprising 148 individuals with COVID-19 and 116 individuals without COVID-19 (**Table 1**). Although the data presentation compares COVID-19-positive and -negative groups, the study was conducted prospectively with no prior matching or retrospective case selection. The mean age was similar between groups (58.11 ± 22.29 years in COVID-positive vs. 57.84 ± 17.47 years in COVID-negative; *p* = 0.57). Likewise, the sex distribution did not differ significantly between the two groups, with males representing 38.5% of COVID-positive patients and 38.8% of COVID-negative patients (*p* = 1.00).

**Table 1:** Clinical and demographic characteristics of the study participants.

Characteristic	All Samples	COVID-19 Status		*P*
		COVID-19 positive (n = 148)	COVID-19 negative (n = 116)	
Characteristic		Mean ± SD	Mean ± SD	
Age (years)	57.9 ± 19.7	58.11 ± 22.29	57.84 ± 17.47	0.57
Sex N(%)				
Males	102 (39%)	57 (39%)	45 (39%)	1
Females	162 (61%)	91 (61%)	71 (61%)	
BMI Category N (%)				
Normal (18.5 – 24.9 kg/m^2^)	110 (42%)	55 (37%)	55 (47%)	0.05*
Overweight (25 – 29.9 kg/m^2^)	101 (38%)	58 (39%)	43 (37%)	
Obese (>30 kg/m^2^)	53 (20)	35 (24%)	18 (16%)	
**Vitamin D (ng/mL)**	25.9 ± 13.8	25.32 ± 13.27	26.51 ± 14.70	0.77
Vitamin D Status N(%)				
Deficiency (<20 ng/mL)	71 (35%)	42 (34%)	28 (34%)	0.95
Insufficiency (20–30 ng/mL)	72 (35%)	40 (33%)	29 (34%)	
Sufficient (>30 ng/mL)	62 (30%)	40 (33%)	26 (32%)	
Vitamin D Supplements N(%)				
No	120 (45%)	69 (47%)	51 (44%)	0.71
Yes	144 (55%)	79 (53%)	65 (56%)	

Data are presented as mean ± standard deviation for continuous variables and as number (percentage) for categorical variables. *P*-values were calculated using independent samples t-tests or Chi-square tests, with *p* ≤ 0.05 considered statistically significant.

Analysis based on BMI categories revealed a significantly lower proportion of COVID-positive patients with a normal BMI (37.2%) compared to those with a negative test result (47.4%; *p* = 0.05). However, the proportions of overweight and obese participants were comparable between groups. Serum 25(OH)D concentrations did not significantly differ between COVID-positive (25.32 ± 13.27 ng/mL) and COVID-negative patients (26.51 ± 14.70 ng/mL; *p* = 0.77). Similarly, vitamin D status categories (deficiency, insufficiency, sufficiency) and vitamin D supplement use showed no significant differences (*p*> 0.05).

Multivariate logistic regression analysis identified age ≥60 years as a significant predictor of increased COVID-19 disease risk (OR = 1.90, 95% CI: 1.02–3.55, *p* = 0.04). Conversely, sex, BMI categories, and vitamin D status did not independently predict COVID-19 disease (*p* > 0.05) (**Table 2**).

**Table 2:** Multivariate logistic regression analysis of predictors of COVID-19 disease: clinical parameters.

Characteristic	COVID-19 disease		
	OR	95% C.I.	*P*
Age			
<60	1		0.04*
≥60	1.90	(1.02 – 3.55)	
Sex			
Male	1		0.25
Female	1.48	(0.77 - 2.76)	
BMI			
Normal (18.5 – 24.9 kg/m^2^)	1		
Overweight (25 – 29.9 kg/m^2^)	0.48	(0.21 – 1.09)	0.08
Obese (>30 kg/m^2^)	0.57	(0.25 – 1.29)	0.18
Vitamin D status			
Deficiency (<20 ng/mL)	1		
Insufficiency (20–30 ng/mL)	0.75	(0.36 – 1.56)	0.44
Sufficient (>30 ng/mL)	0.76	(0.37 – 1.58)	0.45

Odds ratios (OR), 95% confidence intervals (CI), and *p*-values are provided for age, sex body mass index (BMI), and vitamin D status, along with reference categories.

In the subset of 70 patients evaluated for gene expression (**Table 3**), higher *VDR* expression in nasopharyngeal samples was significantly associated with a reduced likelihood of COVID-19 disease (OR = 0.40, 95% CI: 0.15–1.06, *p* = 0.05). Likewise, elevated *DEFA1–3* mRNA expression exhibited strong protective effects, significantly reducing COVID-19 disease risk by 81.6% (OR = 0.184, 95% CI: 0.035–0.97, *p* = 0.012). Conversely, *CCL20* expression did not differ significantly between COVID-positive and COVID-negative patients (*p* = 0.294).

**Table 3:** Multivariate logistic regression analysis with COVID-19 disease predictors: gene expression data.

Characteristics	COVID-19 Disease		
	OR	95% C.I.	*P*
Age			
<60	1		
≥60	5.250	1.547-17.821	0.008*
Sex			
Male	1		
Female	3.359	0.891-12.658	0.073
BMI			
Normal	1		
Overweight	1.151	0.314-4.219	0.832
Obese	1.891	0.490-6.881	0.491
Normalized DEFA1–3 Expression			
Low	1		
High	0.184	0.035-0.960	0.012*
Normalized CCL20 Expression			
Low	1		
High	0.532	0.163-1.732	0.294
Normalized *VDR* Expression			
Low	1		
High	0.4	(0.15 - 1.06)	0.05*

Gene expression levels were classified as low or high based on their median value.

Odds ratios (OR), 95% confidence intervals (CI), and *p*-values for age, sex, BMI, and gene expression levels of defensin alpha 1-3 (*DEFA1-3*), chemokine (C-C motif) ligand 20 (*CCL20*), and vitamin D receptor-1 (*VDR-1*). *indicates statistical significance (*p* ≤ 0.05).

**Figure 1:**
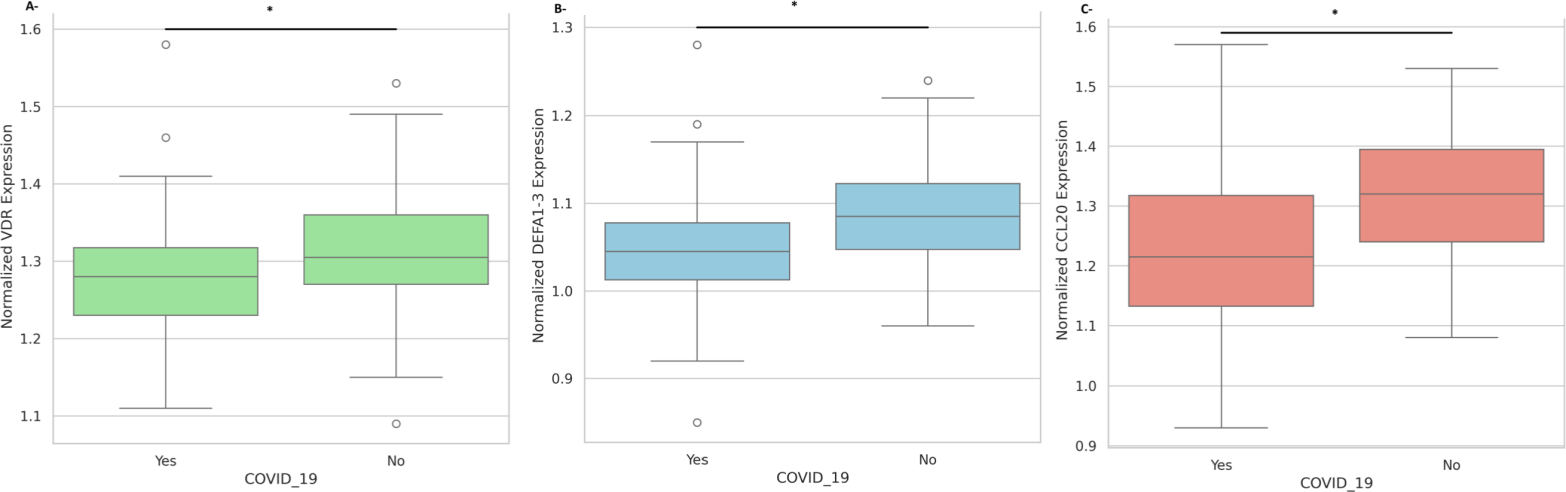
Nasopharyngeal expression of innate immune mediators according to COVID-19 disease status. **(A)** Normalized expression levels of *VDR* mRNA in COVID-19 negative versus COVID-19 positive participants. **(B)** Normalized expression levels of *DEFA1–3* mRNA in COVID-19 negative versus COVID-19 positive participants. **(C)** Normalized expression levels of *CCL20* mRNA in COVID-19 negative versus COVID-19 positive participants. All expression values are normalized to *GAPDH*. Data are presented as mean ± standard deviation. Statistical significance is indicated (*p < 0.05).

The gene expression comparison according to the COVID-19 disease status showed that the normalized *VDR, DEFA1–3 and CCL20* expression is significantly higher in the negative group than in the positive group (**Figure 1**, *P*<0.05).

Pearson correlation analysis demonstrated a significant inverse relationship between serum 25(OH)D concentrations and *VDR* expression (r = -0.61, *p* = 0.04). Interestingly, In the COVID-19 negative group, *VDR* expression was positively correlated with *CCL20* expression (r = 0.59, *p* < 0.01), while no significant correlation was observed between *VDR* and *DEFA1-3* (r = –0.15) or between *CCL20* and *DEFA1-3* (r = –0.04). In the COVID-19 positive group, *VDR* expression showed a significant positive correlation with *DEFA1-3* (r = 0.45, *p* < 0.05). The correlation between *CCL20* and *DEFA1–3* was weak and nonsignificant (r = 0.16) in the positive group (**Table 4**). When pooling samples, *VDR* expression remained significantly correlated with *CCL20* (r = 0.45, *p* < 0.01).

**Table 4:** Pearson correlation analysis of normalized *VDR*, *CCL20*, and *DEFA1–3* expression stratified by COVID-19 disease status.

	*VDR*	*CCL20*	*DEFA1-3*
COVID-19 NEGATIVE (n=40)			
*VDR*	**-**	r = 0.59, *p* < 0.01	r = –0.15, *p *= N.S.
*CCL20*	r = 0.59, *p < 0.01*	**-**	r = –0.04, *p* = N.S.
*DEFA1-3*	r = –0.15, *p* = N.S.	r = –0.04, *p* = N.S.	**-**
COVID-19 POSITIVE (n=30)			
*VDR*	**-**	r = 0.28, *p* = N.S.	r = 0.45, *p* < 0.05
*CCL20*	r = 0.28, *p* = N.S.	–	r = 0.16, *p* = N.S.
*DEFA1-3*	r = 0.45, *p* < 0.05	r = 0.16, *p* = N.S.	**-**
All Samples (n=70)			
*VDR*	**-**	r = 0.45, *p* < 0.01	r = 0.15, *p* = N.S.
*CCL20*	r = 0.45, *p* < 0.01	**-**	r = 0.15, *p* = N.S.
*DEFA1-3*	r = 0.15, *p* = N.S.	r = 0.15, *p* = N.S.	**-**

*N.S., Not Significant.

Correlation analysis among COVID-19-positive patients showed no significant associations between *DEFA1–3* and *CCL20* expression or with SARS-CoV-2 replication genes *(ORF1*, *N*, *E*) (all *p >*0.05). However, strong correlations were observed among the viral replication genes themselves (*ORF1*, *N*, and *E*; all r >0.99, *p <*0.001), validating their use as reliable markers of viral replication (**Supplementary Table 1**).

## Discussion

Our integrated analysis, combining clinical data from a cohort with detailed gene expression profiling in a representative subset, provides critical insights into the roles of vitamin D and innate immune responses in COVID-19 susceptibility. Despite similar serum 25(OH)D concentrations between COVID-19-positive and negative patients, this finding suggests that circulating vitamin D concentrations alone may inadequately reflect the full immunomodulatory potential of vitamin D. Rather, local tissue responsiveness—as represented by *VDR* expression in nasopharyngeal tissues—emerges as a crucial determinant of protective immunity. Higher *VDR* expression correlated with a significant 60% reduction in COVID-19 disease risk, highlighting the importance of receptor-mediated signaling within local mucosal environments.

Additionally, analysis of inflammatory biomarkers indicated that elevated *DEFA1–3* expression significantly reduces COVID-19 susceptibility by 81.6% (OR: 0.184, *p* = 0.012). *DEFA1–3* peptides are known for their potent antiviral activities, including disruption of viral membranes, inhibition of viral entry, and modulation of local immune responses (17, 26). Their protective role was further supported by our finding of higher *DEFA1–3* expression in COVID-19-negative individuals, consistent with reduced viral susceptibility. This aligns with observations by Idris et al. (27), who reported significant downregulation of *DEFA1–3* during active SARS-CoV-2 infection, suggesting a possible viral evasion mechanism through suppression of host antimicrobial peptides. This suppression may partly explain the increased susceptibility to secondary infections observed in severe COVID-19 cases (27).

However, the role of *DEFA1–3* extends beyond direct antiviral activity. Alpha-defensins, including *DEFA1-3*, have complex functions that involve immune modulation and inflammatory responses. Elevated alpha-defensin levels have been associated with thrombotic complications in COVID-19 through interactions with fibrinogen and interleukin-6, highlighting their dual roles in both protective immunity and pathology (28). *DEFA1–3* peptides facilitate neutrophil recruitment and cytokine production, potentially driving beneficial inflammation for pathogen clearance; however, excessive or dysregulated activity could lead to tissue damage and exacerbated pathology (15, 28). This delicate balance underscores the importance of cautious interpretation and further investigation into the role of defensins during SARS-CoV-2 infection.

In our study, *DEFA1–3* expression showed no significant correlations with SARS-CoV-2 viral replication genes (*ORF1*, *N*, and *E*), suggesting that defensin activity operates independently of viral replication dynamics and is likely influenced predominantly by host factors (29). Consequently, *DEFA1–3* expression may serve as a valuable biomarker for assessing susceptibility to COVID-19. While elevated baseline defensin levels may reflect robust innate immunity, they could also signal immune dysregulation or exhaustion (28). Measuring defensin levels clinically enhances risk assessment and patient management strategies.

In contrast to *DEFA1-3*, *CCL20* expression did not differ significantly between infected and non-infected individuals, suggesting variability in the contribution of inflammatory mediators to COVID-19 susceptibility (22–24). However, our results suggest that the role of *CCL20* in disease susceptibility may be context-dependent, warranting further investigation.

The correlation patterns suggest that vitamin D signaling through *VDR* is functionally linked to immune cell recruitment (via *CCL20*) under non-infectious conditions. However, this relationship appears to be disrupted during COVID-19 disease, where vitamin D signaling may shift toward enhancing antimicrobial peptide production (*DEFA1-3*) as part of the host defense mechanism. The loss of *VDR-CCL20* correlation during infection could reflect immune system dysregulation or a shift in immune response priorities under infectious stress.

Our study has several limitations. Although our findings highlight reduced 25(OH)D levels in COVID-19 cases compared to controls, clinical severity data were not collected, precluding stratified analysis. Previous reports have shown that vitamin D status may prospectively predict COVID-19 severity and outcomes (30, 31). The study design limits our ability to establish causality and to assess longitudinal changes in vitamin D status, *VDR* expression, and inflammatory biomarkers. Additionally, although the overall cohort was sizable (n = 264), the subset for gene expression analysis was relatively limited, which may have limited its representativeness and generalizability. Potential confounders, including seasonal variations in vitamin D levels, nutritional status, and other environmental factors, were not fully controlled. Another limitation is the lack of data on participants’ COVID-19 vaccination status, as it was not available at the time of data collection.

Given the high prevalence of vitamin D deficiency in Lebanon and its apparent link to COVID-19 susceptibility, future interventional trials should evaluate the efficacy and optimal dosing of vitamin D supplementation in reducing infection risk and disease severity. We recommend systematic screening for 25(OH)D levels in at-risk populations (e.g., individuals with limited sun exposure) followed by randomized controlled studies to determine whether correcting deficiency can improve clinical outcomes in SARS-CoV-2 infection.

## Conclusion

**Figure 2:**
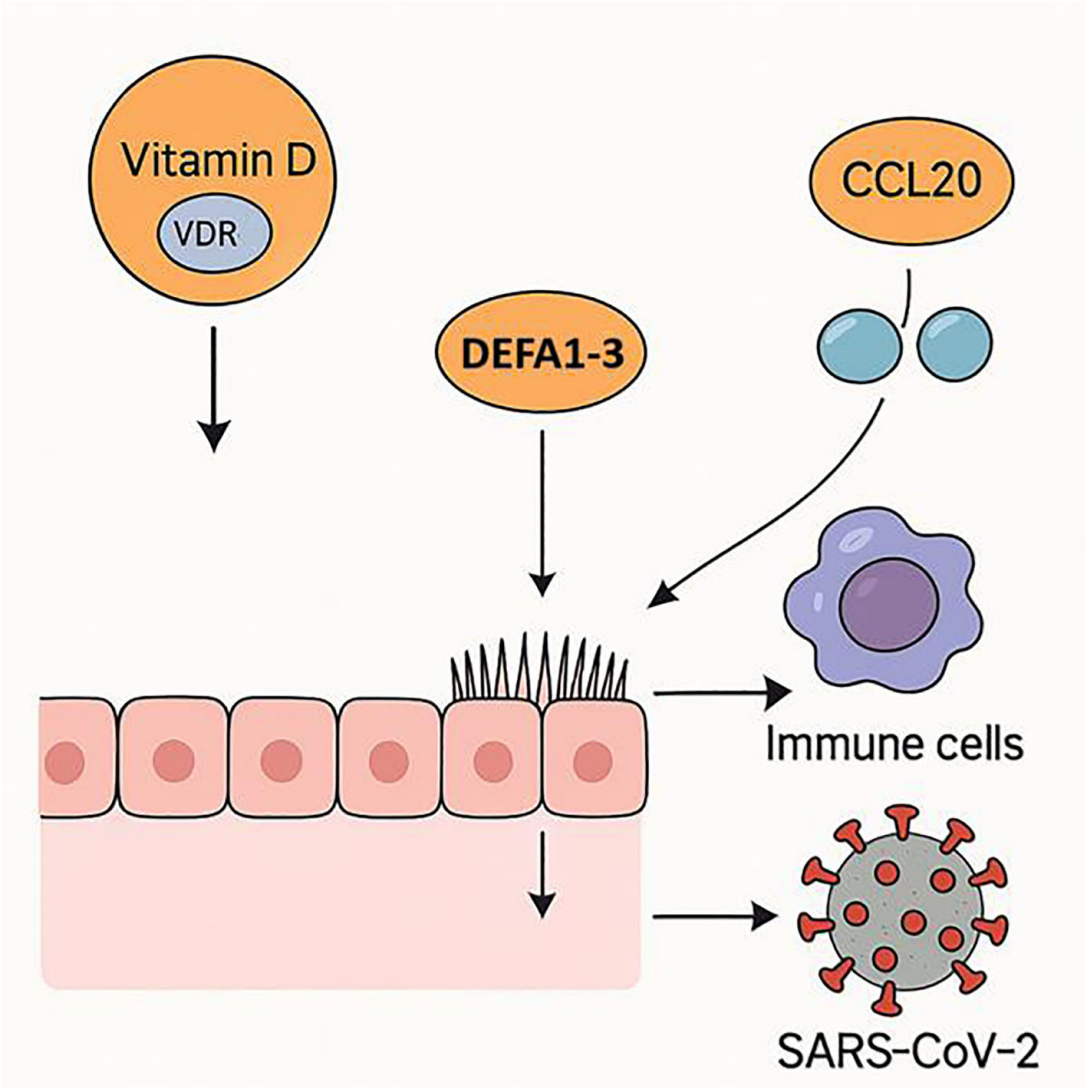
Proposed mechanism of vitamin D in reducing SARS-CoV-2 susceptibility. Vitamin D signaling via *VDR* in respiratory epithelium enhances antimicrobial defenses through *DEFA1–3* upregulation and facilitates immune cell recruitment by modulating *CCL20* expression, collectively lowering the risk of SARS-CoV-2 infection.

In summary, our findings demonstrate that the protective effects of vitamin D in COVID-19 are more closely associated with local *VDR* expression and innate antimicrobial pathways, particularly DEFA1-3, than with systemic 25(OH)D concentrations alone (**Figure 2**). These results support a model in which both micronutrient status and tissue-specific vitamin D signaling modulate host susceptibility and disease severity (**Figure 2**). Future longitudinal and interventional studies are warranted to validate these associations and explore the therapeutic potential of enhancing local vitamin D responsiveness in respiratory viral infections.

## Data Availability

The original contributions presented in the study are included in the article/**Supplementary Material**, further inquiries can be directed to the corresponding author/s.
