# Supplementary material for: Evaluation of vitamin D status, vitamin D receptor expression, and innate immune mediators in COVID-19
Source: Front Endocrinol (Lausanne). 2025 Aug 19;16:1600623. doi: 10.3389/fendo.2025.1600623 (PMC12401688; doi:10.3389/fendo.2025.1600623)
Supplement: Supplementary file 2 [file Table1.docx]

**Supplementary Table 1. Pearson Correlation Coefficients Among Gene Expression Levels.**

|  | ***DEFA1-3*** | ***CCL20*** | ***ORF1*** | ***E* gene** | ***N* gene** |
| --- | --- | --- | --- | --- | --- |
| ***DEFA1-3*** | - | 0.161 | 0.244 | -0.233 | -0.213 |
| ***CCL20*** | 0.161 | - | 0.212 | 0.208 | 0.213 |
| ***ORF1*** | 0.244 | 0.212 | - | 0.995 ** | 0.992 ** |
| ***E* gene** | -0.233 | 0.208 | 0.995 ** | - | 0.995 ** |
| ***N* gene** | -0.213 | 0.213 | 0.992 ** | 0.995 ** | - |

Correlation coefficients among defensin alpha 1-3 (*DEFA1-3*), chemokine (C-C motif) ligand 20 (*CCL20*), open reading frame 1 (*ORF1*), envelope (*E*) gene, and nucleocapsid (*N*) gene in COVID-19 positive patients are presented. ** indicates p < 0.001.
